# Dexamethasone exposure during pregnancy triggers metabolic syndrome in offspring *via* epigenetic alteration of IGF1

**DOI:** 10.1186/s12964-024-01472-6

**Published:** 2024-01-23

**Authors:** Hao Xiao, Bo He, Heze Liu, Yawen Chen, Di Xiao, Hui Wang

**Affiliations:** 1 Department of Pharmacology, Wuhan University School of Basic Medical Sciences, Wuhan, 430071 China; 2 Division of Joint Surgery and Sports Medicine, Department of Orthopedic Surgery, Zhongnan Hospital of Wuhan University, Wuhan, 430071 China; 3 Hubei Provincial Key Laboratory of Developmentally Originated Disease, Wuhan, 430071 China; 4 School of Pharmaceutical Sciences & Yunnan Key Laboratory of Pharmacology for Natural Products/College of Modern Biomedical Industry, NHC Key Laboratory of Drug Addiction Medicine, Kunming Medical University, Kunming, 650500 China

**Keywords:** Prenatal dexamethasone exposure, Metabolic syndrome, Endogenous glucocorticoids, Insulin-like growth factor 1, Histone acetylation

## Abstract

**Background:**

Previous research has reported that prenatal exposure to dexamethasone (PDE) results in organ dysplasia and increased disease susceptibility in offspring. This study aimed to investigate the epigenetic mechanism of metabolic syndrome induced by PDE in offspring.

**Methods:**

Pregnant Wistar rats were administered dexamethasone, and their offspring’s serum and liver tissues were analyzed. The hepatocyte differentiation model was established to unveil the molecular mechanism. Neonatal cord blood samples were collected to validate the phenomenon and mechanism.

**Results:**

The findings demonstrated that PDE leads to insulin resistance and typical metabolic syndrome traits in adult offspring rats, which originated from fetal liver dysplasia. Additionally, PDE reduced serum corticosterone level and inhibited hepatic insulin-like growth factor 1 (IGF1) signaling in fetal rats. It further revealed that liver dysplasia and functional impairment induced by PDE persist after birth, driven by the continuous downregulation of serum corticosterone and hepatic IGF1 signaling. Both in vitro and in vivo experiments confirmed that low endogenous corticosterone reduces the histone 3 lysine 9 acetylation (H3K27ac) level of IGF1 and its expression by blocking glucocorticoid receptor α, special protein 1, and P300 into the nucleus, resulting in hepatocyte differentiation inhibition and liver dysplasia. Intriguingly, neonatal cord blood samples validated the link between reduced liver function in neonates induced by PDE and decreased serum cortisol and IGF1 levels.

**Conclusions:**

This study demonstrated that low endogenous glucocorticoid level under PDE lead to liver dysplasia by downregulating the H3K27ac level of IGF1 and its expression, ultimately contributing to metabolic syndrome in adult offspring.

**Supplementary Information:**

The online version contains supplementary material available at 10.1186/s12964-024-01472-6.

## Background

Metabolic syndrome is a health condition characterized by elevated lipid levels, low levels of high-density lipoprotein-cholesterol (HDL-C), high blood pressure, disrupted glucose regulation, and obesity. This condition increases the risk of developing type 2 diabetes, cardiovascular disease, and atherosclerosis [1]. Additionally, insulin resistance is recognized as a central feature of metabolic syndrome [2]. Studies have shown that individuals with low birth weight are more prone to metabolic syndrome in adulthood, suggesting that metabolic syndrome has fetal origin [3–5]. Adverse conditions during pregnancy can result in intrauterine growth retardation (IUGR), characterized by low birth weight and dysfunction of multiple organs [6–8]. Although dexamethasone is used to promote fetal lung maturation and reduce neonatal respiratory distress syndrome, its use during pregnancy is an adverse factor for fetal development [9]. Researches have demonstrated that fetuses exposed to dexamethasone during pregnancy often have low birth weight, with birth weight negatively correlated with the duration of dexamethasone therapy [10–12]. Our previous animal studies confirmed that prenatal dexamethasone exposure (PDE) led to low birth weight in fetal offspring, contributing to diseases in adulthood, including osteoporosis, epilepsy, and glomerulosclerosis [13–15]. However, it remains unclear whether PDE can induce metabolic syndrome in offspring, whether it originates from intrauterine liver dysplasia, and what the specific epigenetic programming mechanism is.

The liver, an essential metabolic organ, plays a crucial role in regulating glucose and lipid metabolism. Liver development is a complex process that involves the formation of hepatic progenitor cells and the differentiation of hepatocytes [16, 17]. The maturation of hepatocytes can be assessed by examining the expression of hepatocyte-specific genes like albumin (ALB) and alpha-fetoprotein (AFP) [18, 19]. The insulin-like growth factor 1 (IGF1) signaling pathway is a central component of the endocrine regulatory system, influencing the proliferation, differentiation, and metabolism of various cells before and after birth [20–22]. Fetal blood IGF1 primarily originates from the fetal liver, highlighting its critical role in liver development. In normal circumstances, basal levels of endogenous glucocorticoids (cortisol in humans and corticosterone in rodents) are responsible for promoting fetal development [23]. However, it is unclear whether low levels of endogenous blood glucocorticoids can impact fetal liver development in offspring exposed to prenatal dexamethasone by regulating hepatic IGF1 expression.

Both human and animal studies provide compelling evidence that adverse intrauterine conditions can result in permanent changes to fetal tissue structure and function, affecting development into adulthood. This phenomenon is referred to as “intrauterine programming” [24]. Previous research has highlighted the potential role of epigenetic modifications in programming phenotypic changes during fetal development [25]. For instance, histone acetylases can influence the expression of genes related to development through histone acetylation. In this study, using a rat model, hepatocyte differentiation cells, and clinical blood specimens, we aimed to investigate the impact of PDE on liver development in offspring and its connection to metabolic syndrome in adulthood. Additionally, we sought to unravel the epigenetic programming mechanisms involved. This research holds practical significance for guiding the judicious use of dexamethasone during pregnancy in clinical settings and exploring early prevention and treatment strategies for metabolic syndrome with fetal origins. Furthermore, it contributes to the understanding of the “Developmental Origin of Health and Disease (DOHaD)” theory.

## Methods

### Drugs and reagents

Dexamethasone was sourced from Shuanghe Pharmaceutical Company in Wuhan, China. We used enzyme-linked immunosorbent assay (ELISA) kits for rat corticosterone and human cortisol from Assaypro in Saint Charles, MO, USA. The ELISA kit for serum IGF1 concentration (No. MG100) was procured from R&D Systems, Inc. in Minneapolis, MN, USA. Overexpression plasmids for glucocorticoid receptor α (GRα), special protein (SP) 1, and P300 were obtained from GenePharma in Shanghai, China. TRIZOL was purchased from Invitrogen Co. in Carlsbad, CA, USA. Reverse transcription and real-time quantitative polymerase chain reaction (RT-qPCR) kits were procured from TaKaRa Biotechnology Co., Ltd in Dalian, China. Insulin was purchased from Jiangsu Wanbang Biochemical Pharmaceutical Group Co., Ltd, China (No. 22,212,216). All primers were synthesized by Sangon Biotech Co., Ltd. in Shanghai, China. The SYBR Green dye was purchased from Applied Biosystems by Thermo Fisher Scientific (ABI) in Foster City, CA, USA. The antibody for Ki67 (No. ab15580) was obtained from Abcam plc. in Cambridge, UK. Anti-rabbit glyceraldehyde-3-phosphate dehydrogenase (GAPDH) (No. 2118) and Histone 3 (No. 9715) were sourced from Cell Signaling in Massachusetts, USA. The antibodies for GRα (sc-376,426) and P300 (sc-48,343) were procured from Santa Cruz Biotech Co. in Santa Cruz, CA, USA. The antibody for SP1 (ab227383) was obtained from Abcam plc. in Cambridge, UK. Antibodies for histone 3 lysine 9 acetylation (H3K9ac) (A7255), H3K14ac (A7254), and H3K27ac (A7253) were sourced from ABclonal Biotech Co., Ltd. in Wuhan, China. All other chemicals and reagents used were of analytical grade.

### Animal experimental procedure

**Fig. 1:**
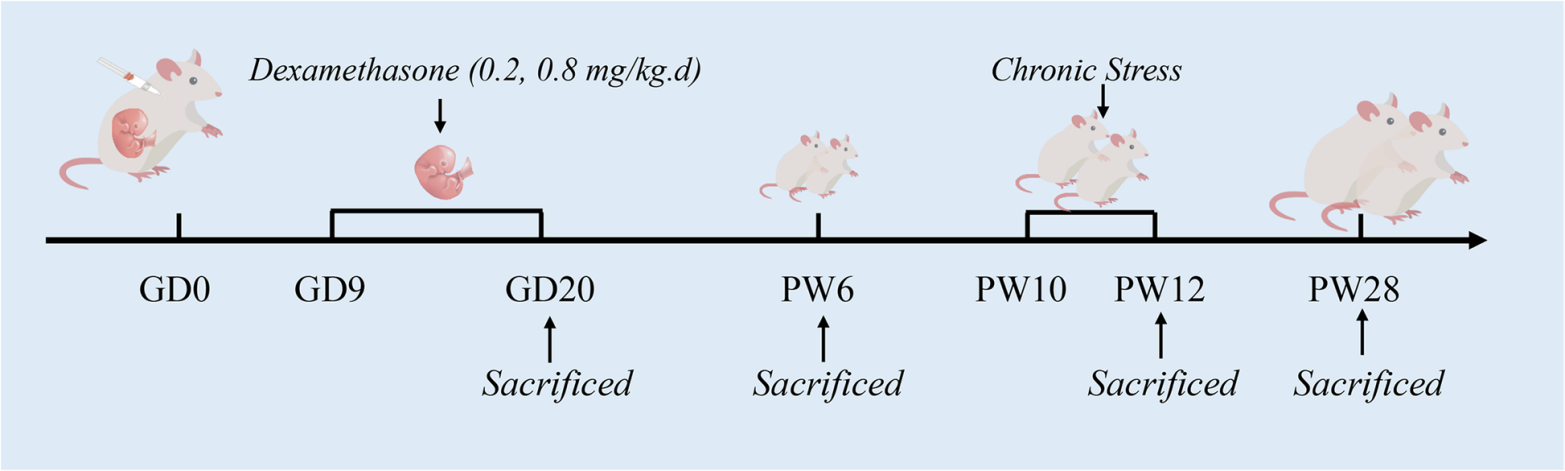
Animal experimental procedure. GD, gestational day; PW, postnatal week

Animal experiments were conducted in accordance with rigorous ethical and accreditation standards. Specifically, they were carried out at the Center for Animal Experiment of Wuhan University in Wuhan, China, an institution accredited by the Association for Assessment and Accreditation of Laboratory Animal Care International (AAALAC International). All procedures involving animals followed the Guidelines for the Care and Use of Laboratory Animals established by the Chinese Animal Welfare Committee. The Committee on the Ethics of Animal Experiments at the Wuhan University School of Medicine granted approval for the animal experimental protocol under Permit No. 201,709. These animal studies adhered to the ARRIVE guidelines. We used specific pathogen-free adult Wistar rats, with females weighing 200 ± 20 g and males weighing 280 ± 20 g, which were procured from the Experimental Center of the Hubei Medical Scientific Academy in Wuhan, China. These rats were kept in a controlled environment with a temperature of 18–22 °C, humidity ranging from 40 to 60%, and a 12-hour light-dark cycle. They were provided with free access to food and water. To initiate mating, two female rats were placed together with one male rat overnight in a cage. The appearance of sperm in a vaginal smear marked the onset of gestational day (GD) 0. The mating success rate is approximately 60%. Subsequently, pregnant rats were individually housed and randomly allocated into the control and PDE groups, as illustrated in Fig. 1. To minimize bias in the animal experiments, rats were housed and administered treatments by a technician. Different co-authors were responsible for sample collection and data analysis, respectively.

The animal experiment during pregnancy: Pregnant rats were transferred to individual cages and then randomly divided into the control, PDE(L) and PDE(H) groups. From GD9 to GD20, animals from the PDE(L) group were subcutaneously injected 0.2 mg/kg∙d dexamethasone in the neck scruff, and animals from the PDE(H) group were subcutaneously injected 0.8 mg/kg∙d dexamethasone in the neck scruff. In contrast, the control group was administered an equivalent volume of distilled water. On GD20, the pregnant rats were anesthetized with isoflurane, and their fetuses were extracted *via* laparotomy. The fetal rats’ body weight and sex were documented. Fetal rats weighing less than 2 standard deviations from the control group’s average body weight were categorized as intrauterine growth-restricted (IUGR) fetal rats. Fetal rat serum was collected and pooled within each litter, then stored at −80 °C for subsequent analysis. Fetal livers were stored in a −80 °C deep freezer for further examination of mRNA and protein expression, or fixed in 4% paraformaldehyde for morphological staining and immunohistochemistry.

The animal experiment in the postnatal period: The pregnant rats, comprising both the control group and PDE group (0.2 mg/kg.d), delivered their offspring naturally. The day of birth was designated as postnatal day (PD) 0. To ensure adequate nourishment of the offspring, litters with 10–14 offspring rats were selected from each group on PD1. The number of male and female rats was adjusted to six in each litter for nursing and feeding. One of the two male pups underwent a 2-week ice-water swimming test (4–8 °C for 5 min per day), starting at postnatal week (PW) 10. One hour after the final swim, these rats were humanely euthanized under isoflurane anesthesia at PW12, with eight individuals selected from eight different litters. The other male offspring rats were fed a regular diet until PW28 after weaning. Intraperitoneal glucose tolerance tests (IPGTT) and insulin tolerance tests (ITT) were conducted at PW28, followed by euthanasia under isoflurane anesthesia. Serum and tissues were gathered for subsequent analysis of mRNA and protein expression or fixed in 4% paraformaldehyde for hematoxylin-eosin (H&E) staining and immunohistochemistry.

### Human subjects and cord blood specimens were collected

This study was granted approval by the Human Research Committee of Zhongnan Hospital at Wuhan University (No. 2,016,016). For this research, medical records of pregnant women and their newborns from January 1, 2019, to March 20, 2022, were utilized. Inclusion criteria were as follows: ① Women received prenatal dexamethasone therapy (PDT) or had no dexamethasone therapy between gestational weeks 34 to 42 and gave birth within 24 h to 7 days; ② These pregnant women had singleton pregnancies. Exclusion criteria included: ① Pregnant women with comorbidities such as hypertension, diabetes, heart disease, autoimmune diseases, and other chronic conditions; ② The use of other drugs, such as the synthetic glucocorticoid prednisone. At the time of delivery, umbilical cord blood from neonates was collected to further analyze serum levels of cortisol, IGF1, ALB, and AFP in both groups.

### Bone marrow mesenchymal stem cells (BMSCs) extraction and hepatoid differentiation

BMSCs were derived from 4-week-old male rats. Briefly, the rats were sacrificed and immersed in 75% ethanol for 10 min. Subsequently, femurs and tibias were aseptically separated. Both ends of the epiphyses were sterilely removed, and the bone marrow cavity was flushed with a 5 mL syringe containing α-minimum essential medium (α-MEM). The thoroughly dispersed flush was then filtered through a 200-mesh nylon filter, and the resulting supernatant was centrifuged at room temperature at 1000 r/min for 10 min. Following centrifugation, the cells were collected and seeded at a density of 4 × 10^5^ cells per well in six-well plates with growth medium (α-MEM with 10% fetal bovine serum, 100 mg/mL streptomycin, and 100 U/mL penicillin). Upon reaching 80% confluence, the growth medium was switched to hepatocyte differentiation medium (low-glucose DMEM medium supplemented with 1% fetal bovine serum, 100 mg/mL streptomycin, 100 U/mL penicillin, 20 ng/mL hepatocyte growth factor, 2 ng/mL epidermal growth factor, 0.1 µM dexamethasone, and 50 mg/mL insulin-transferrin-selenium) for a duration of 14 days. Subsequently, the cells were exposed to various concentrations of corticosterone (250, 125, 62.5 nM) for 3 days, after which they were harvested for further analysis.

### IPGTT, ITT and IRI

For IPGTT, rats fasted overnight for 12 h and were intraperitoneally injected with 2 g/kg glucose solution. The serum samples were collected by tail vein clipping at 0, 15, 30, 60, and 120 min after administration. The serum glucose values at each time point were detected by real-time serum glucose test strips, the change curve was drawn and the area under curve (AUC) was calculated. The trapezoidal rule was used to calculate AUC, and the specific formula was as follows:

$$\text{A}\text{U}\text{C}=\frac{{(C}_{0}+{C}_{15})}{2}\times {Time}_{0-15}+\frac{{(C}_{15}+{C}_{30})}{2}\times {Time}_{15-30}+\frac{{(C}_{30}+{C}_{60})}{2}\times {Time}_{30-60}+\frac{{(C}_{60}+{C}_{120})}{2}\times {Time}_{60-120}$$

$C_{X:\:}$Serum glucose values at each time point (mmol/L); time time interval (min).

For ITT, the rats were fasted for 6 h and injected intraperitoneally with 0.75 U/kg short-acting insulin. The serum samples were obtained from the tail vein at 0, 15, 30, 60, and 120 min. Serum glucose at each time point was detected by real-time serum glucose test strips. Serum glucose values at each time point were standardized according to 0 min, the percentage change curve of blood glucose was drawn and AUC was calculated using the same method as above.

### Serum samples analysis

For serum biochemical analysis, rats fasted overnight for 12 h and then sacrificed for serum collection. The levels of serum glucose, insulin, total cholesterol (TCH), triglyceride (TG), low-density lipoprotein-cholesterol (LDL-C), and HDL-C were detected by biochemical assay kits following the manufacturer’s protocol (Shanghai Rongsheng Biotech Co., Ltd., China). Serum corticosterone and IGF1 concentrations were detected by ELISA kit based on the following manufacturer’s instructions provided (Beyotime Biotechnology Co., Ltd., China). Insulin resistance index (IRI) was calculated using the method as follows [26].

$$\mathrm{IRI}=\frac{\mathrm{Fasting}\;\mathrm{glucose}\;\left(\mathrm{mmol}/\mathrm L\right)\;\times\;\mathrm{Fasting}\;\mathrm{insulin}\;\left(\mathrm{{\mu}UI}/\mathrm{mL}\right)}{22.5}$$

### Histological and immunohistochemistry analysis

To explore morphology in liver samples, the 6-µm thickness paraffin histological sections were prepared and routinely stained with hematoxylin and eosin (H&E). The sections were observed and photographed with an Olympus AH-2 light microscope (Olympus, Tokyo, Japan). Eight H&E sections of each group were selected and five random fields of each section were scored under the microscope. The immunohistochemical (IHC) staining for Ki67 of liver tissues was assessed through the routine IHC streptavidin-peroxidase-conjugated method. Sections approximately 6-µm thick were incubated with antibodies specific to Ki67 (diluted 1:200). At least five random fields from each section were examined and analyzed using the Photo Imaging System (Nikon H550S, Japan).

### Overexpression of GR, SP1, and P300 in BMSCs

Before transfection, BMSCs were seeded in six-well plates at a density of 4 × 10^5^ cells per well. Twenty-four hours later, the cells were then transfected respectively with pcDNA3.1.GR, pcDNA3.1.SP1 or pcDNA3.1.p300 vector, empty pcDNA3.1 vector, or empty control vector using Lipofectamine 3000 according to the manufacturer’s protocol. Eight hours later, the medium was exchanged for a fresh medium, and the cells were treated with 250 or 62.5 nM corticosterone. The cells were harvested after 3 days for further analysis.

### Total RNA extract and RT-qPCR

The total RNA was extracted from liver tissues and BMSCs (5 × 10^6^ cells) using the TRIZOL reagent following the manufacturer’s protocol. The tissues of each littermate were pooled for homogenization as one sample. The concentration and purity of the isolated total RNA were determined by a spectrophotometer (NanoDrop 2000 C, Thermo), and the total RNA concentration was adjusted to 1 µg/µL. Then, the total RNA was reverse transcribed using a first-strand cDNA synthesis kit. The RT-qPCR was performed using the ABI Step One RT-PCR thermal cycler (ABI Stepone, USA) with 40 cycles. To quantify the gene transcripts more precisely, the mRNA level of the housekeeping-gene GAPDH was measured and used as a quantitative control. The sequences of each of the designed primers used in this study were shown in Table S1 (Supplementary file).

### Western blotting

Western blotting was performed using a previously described protocol [27]. Briefly, liver tissues and cell samples were obtained. The protein concentration was determined using bicinchoninic acid (BCA) assay. 20 µg protein samples were mixed with 5 × SDS loading buffer, incubated at 95 °C for 5 min, and separated by electrophoresis on 10% SDS-polyacrylamide gels. Proteins were then transferred to polyvinylidene difluoride (PVDF) membranes (EMD Millipore, MA, USA). Membranes were blocked in 5% non-fat milk (26 °C, 60 min) and then incubated (4 °C, overnight) with IGF1 antibody (1:1000), GRα antibody (1:500), SP1 antibody (1:5000), p300 antibody (1:1000), GAPDH antibody (1:5000) and Histone3 antibody (1:5000). Then HRP-conjugated secondary antibody (Santa-Cruz, 1:2000) was incubated and visualized by chemiluminescence using an ECL detection kit (Zhongshan Golden Bridge Biotechnology Co. Ltd., Beijing, China). The results were quantified using Quantity One 1-Analysis software (Bio-Rad Laboratories, Inc.).

### Chromatin immunoprecipitation (ChIP) assay

Cell suspensions from liver tissue and BMSCs (5 × 10^6^ cells) were collected and fixed in 1% formaldehyde for chromatin cross-linking and 125 mM of glycine was added to stop the reaction. The samples were then centrifuged and resuspended in 0.5 ml of lysis buffer containing protease inhibitors. Cell lysates were sonicated to shear DNA to lengths of approximately 200 base pairs and transferred to a new tube with a ChIP dilution buffer. Chromatin was incubated overnight at 4 °C on a nutator/rocker with specific antibodies for H3K9ac (1:50 dilution), H3K14ac (1:100 dilution), or H3K27ac (1:50 dilution), and BSA-treated Protein G beads to reduce nonspecific background binding. The immunoprecipitated DNA-protein complex with beads was collected by centrifugation and washed sequentially with low-salt, high-salt, LiCl immune complex, and Tris-EDTA washing buffer solutions. Freshly prepared elution buffer (1% SDS, 0.1 M NaHCO_3_) was used to elute the DNA protein complex. The samples were then placed in 65 °C water baths overnight to reverse formaldehyde cross-linking and subsequently purified using DNA purification kits (No. DP214-03, Tiangen Biotech Co., Ltd.). The isolated DNA was then assayed using RT-qPCR. Gene enrichment was quantified relative to input controls by RT-qPCR using primers specific to the promoter regions of IGF1.

### Statistical analysis

SPSS 20.0 (SPSS Science Inc., Chicago, Illinois, USA) and Prism 9.0 (GraphPad Software, La Jolla, CA, USA) were used to analyze experimental data. All data were expressed as mean ± standard error of the mean (S.E.M.). The declared group size was the number of independent values, and statistical analysis was done using these independent values. The student’s two tailed *t*-test was performed on one factor of prenatal dexamethasone treatment. For the data from multigroup studies, a post hoc Dunnett-*t*-test or a post hoc Bonferroni–*t*-test was conducted if *F* in one-way analysis of variance (ANOVA) (or equivalent) achieved the chosen necessary level of statistical significance and there was no significant variance inhomogeneity. Pearson correlation analysis was used to analyze the correlation between two indicators. Data of this study are available. A value of *P* < 0.05 was considered statistically significant.

## Results

### PDE caused insulin resistance and metabolic syndrome phenotype in adult male offspring rats

**Fig. 2:**
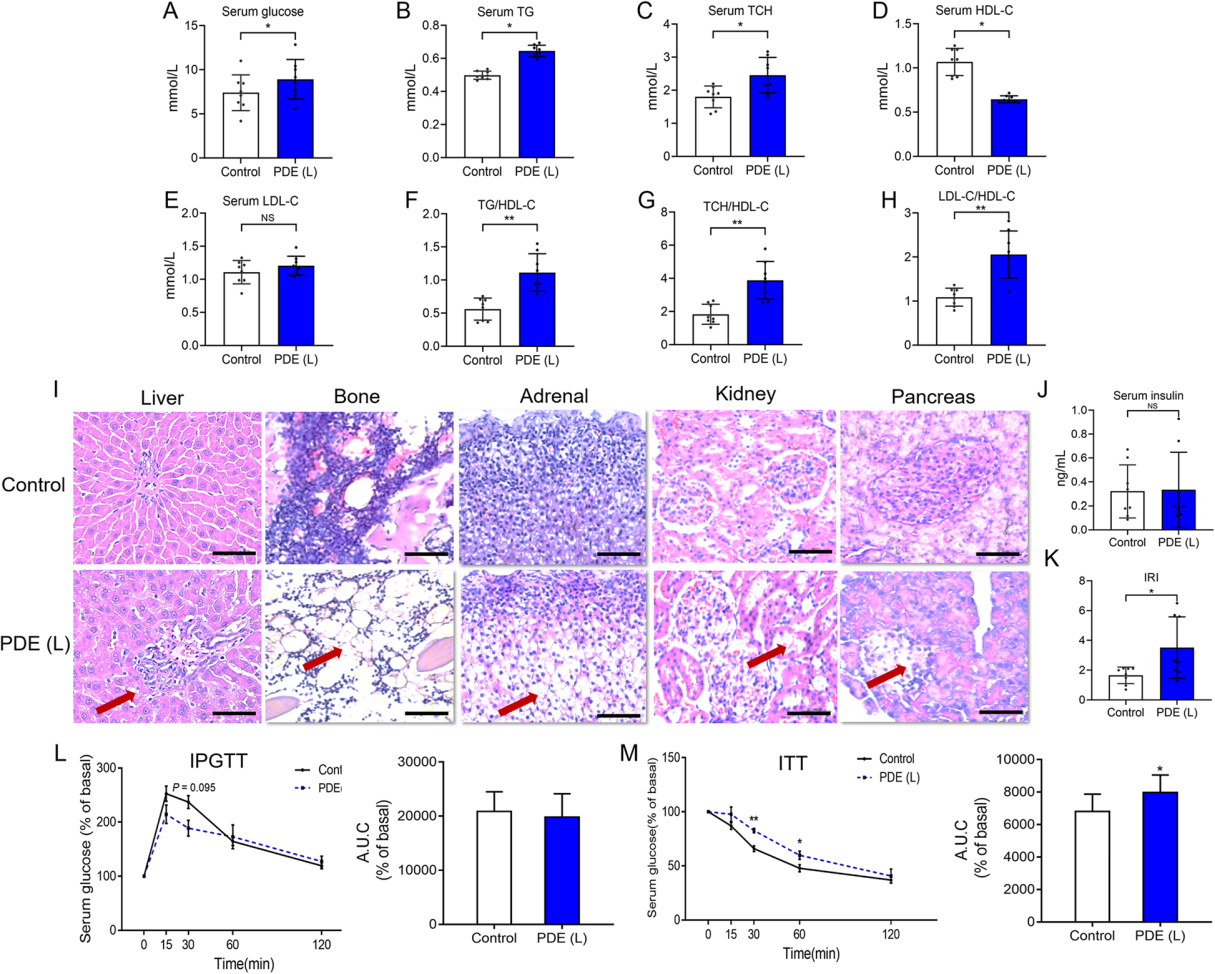
Effects of prenatal dexamethasone exposure (PDE) on glucose and lipid metabolism in the adult male offspring rats at postnatal week 28. **A** Serum glucose level (*n* = 8); (**B**) Serum triglyceride (TG) level (*n* = 8); (**C**) Serum total cholesterol (TCH) level (*n* = 8); (**D**) Serum high-density lipoprotein-cholesterol (HDL-C) level (*n* = 8); (**E**) Serum low-density lipoprotein-cholesterol level (*n* = 8); (**F**) The ratio of TG to HDL-C levels (*n* = 8); (**G**) The ratio of TCH to HDL-C levels (*n* = 8); (**H**) The ratio of LDL-C to HDL-C levels (*n* = 8); (**I**) Histological analysis of multiple organs (Scale bar = 500 μm); (**J**) Serum insulin level (*n* = 8); (**K**) Insulin resistance index (IRI) (*n* = 8); (**L**) Intraperitoneal glucose tolerance test (IPGTT) and the area under curve (AUC) (*n* = 8); M. Insulin tolerance test (ITT) and AUC (*n* = 8). Mean ± S.E.M. ^*^ *P <* 0.05, ^**^ *P <* 0.01 vs. the control group. NS, no significance; PDE(L), prenatal dexamethasone exposure at a low dose (0.2 mg/kg∙d)

First, we investigated the effect of PDE on glucose and lipid metabolism in adult male offspring rats at PW28. The findings revealed that PDE significantly raised serum glucose levels compared to the control group (*P <* 0.05; Fig. 2A). Additionally, PDE significantly elevated serum TG and TCH levels (*P <* 0.05; Fig. 2B and C) while decreasing HDL-C levels (*P <* 0.05; Fig. 2D) in male adult offspring rats. Although there were no significant changes in the serum LDL-C level (Fig. 2E), the ratios of LDL-C/HDL-C, TG/HDL-C, and TCH/HDL-C were all significantly increased by PDE (*P <* 0.01; Fig. 2F-H). Furthermore, we observed that PDE caused lipid accumulation in multiple organs (such as the liver, bone, adrenal gland, kidney, and pancreas) in adult male offspring rats (Fig. 2I). These results indicated that PDE induced classic features of metabolic syndrome in these male adult offspring rats, including hyperglycemia, hyperlipidemia, hypercholesterolemia, and lipid accumulation in various organs.

Given that insulin resistance is a key risk factor for the development of metabolic syndrome [28], we further examined markers related to insulin resistance in these adult male offspring. The results showed that, although PDE did not affect serum insulin levels (Fig. 2J), it significantly increased the IRI (*P <* 0.05; Fig. 2K). Moreover, the results of IPGTT indicated that while there was no significant difference in the initial serum glucose (0 min) value between the control and PDE groups, the serum glucose exhibited a decreasing trend at 15 and 30 min after glucose administration in the PDE group, accompanied by a reduced AUC (Fig. 2L). Additionally, the results of ITT showed that serum glucose levels at 30 and 60 min were significantly increased by PDE, along with an increased AUC (*P <* 0.05, *P* < 0.01; Fig. 2M). This suggests that PDE induced insulin resistance in these adult male offspring rats. In summary, PDE led to insulin resistance and the development of metabolic syndrome in adult male offspring rats.

### The low level of corticosterone and IGF1 participated in liver dysplasia induced by PDE in male fetal rats

**Fig. 3:**
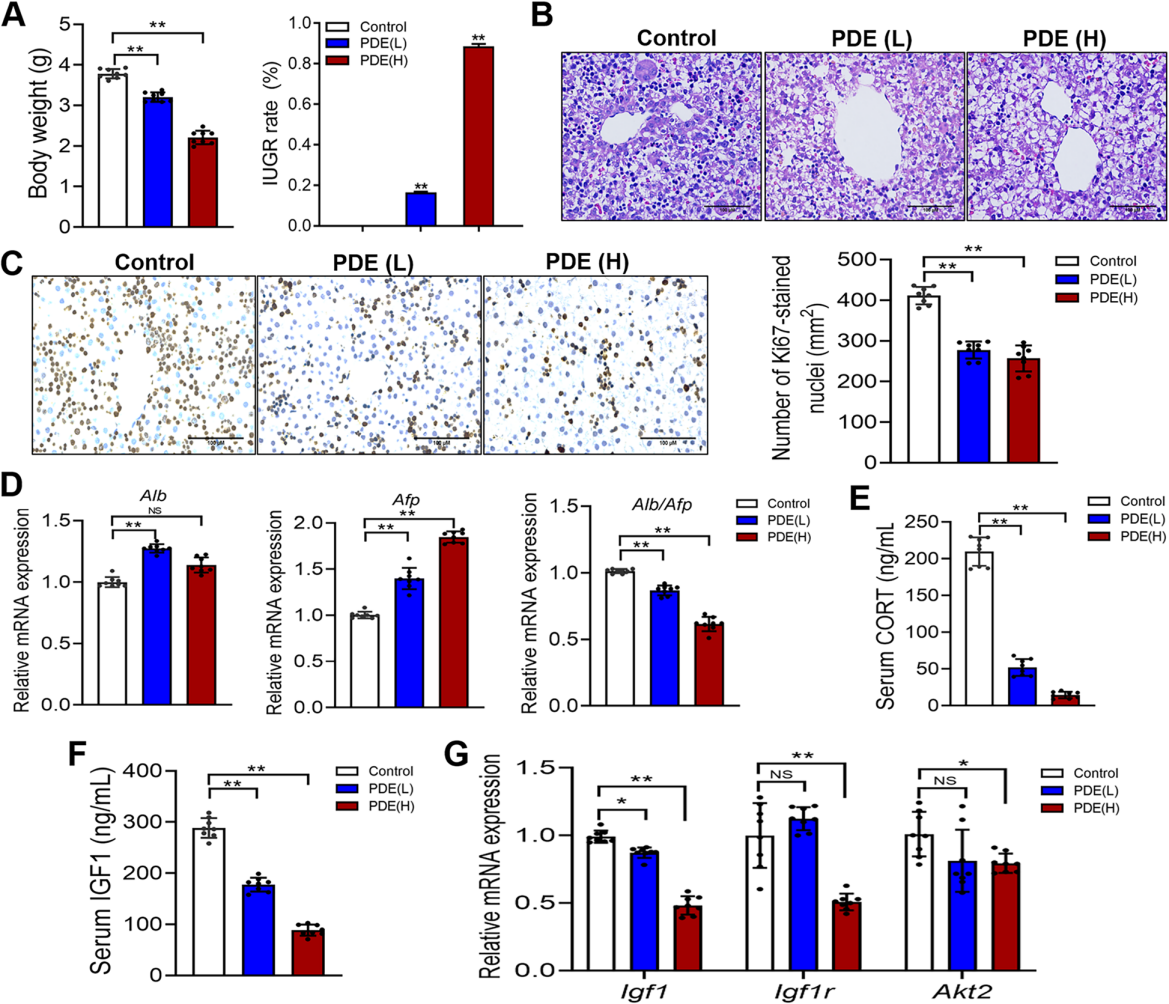
Effects of prenatal dexamethasone exposure (PDE) on the fetal liver development and the levels of corticosterone (CORT) and insulin-like growth factor 1(IGF1). **A** Fetal body weight and intrauterine growth retardation (IUGR) rate (*n* = 8); (**B**) Hematoxylin and eosin (H&E) staining of the fetal liver (Scale bar = 500 μm); (**C**) Ki67-stained nuclei and quantitative analysis (Scale bar = 500 μm); (**D**) The mRNA expression of hepatic development-related genes (*n* = 8); (**E**) Serum CORT level (*n* = 8); (**F**) Serum IGF1 level (*n* = 8); (**G**) The mRNA expression of IGF1 signaling pathway (*n* = 8). Mean ± S.E.M. ^*^ *P <* 0.05, ^**^ *P <* 0.01 vs. the control group. NS, no significance; PDE(L), prenatal dexamethasone exposure at a low dose (0.2 mg/kg∙d); PDE(H), prenatal dexamethasone exposure at a high dose (0.8 mg/kg∙d)

To determine whether the metabolic syndrome induced by PDE originated from intrauterine liver dysplasia, we investigated the effects of PDE on structural and functional development of the fetal liver in male rats. It was observed that PDE reduced body weight and increased the rate of IUGR in male fetal rats in a dose-dependent manner (*P <* 0.01; Fig. 3A). Moreover, H&E staining revealed a vacuole-like phenotype in hepatocytes of the fetal liver exposed to PDE (Fig. 3B). Additionally, the number of Ki67-positive cells, indicating proliferative activity, decreased in a dose-dependent manner in the fetal liver due to PDE (*P <* 0.01; Fig. 3C). Furthermore, we examined the gene expression related to fetal liver development, including *Alb* and *Afp*. The results showed that PDE significantly increased the expression of *Afp*, an inhibitor of liver development, while reducing the expression ratio of *Alb* to *Afp* (*P <* 0.05, *P <* 0.01; Fig. 3D). These findings indicated that PDE caused liver dysplasia in male fetal rats.

Additionally, we analyzed the levels of serum corticosterone and IGF1 in fetal rats. It was observed that PDE led to a dose-dependent reduction in the serum corticosterone level in male fetal rats (*P <* 0.01; Fig. 3E). Simultaneously, there was a decrease in the serum IGF1 level, as well as a decrease in liver *Igf1* expression and its downstream signaling pathway in the fetal rats of the PDE group (*P <* 0.05, *P <* 0.01; Fig. 3F and G). These results collectively indicate that the low-expression programming of corticosterone and IGF1 played a role in the liver dysplasia induced by PDE in male fetal rats.

### The low level of corticosterone caused liver dysplasia in the postnatal male offspring rats of PDE through downregulating IGF1 expression

**Fig. 4:**
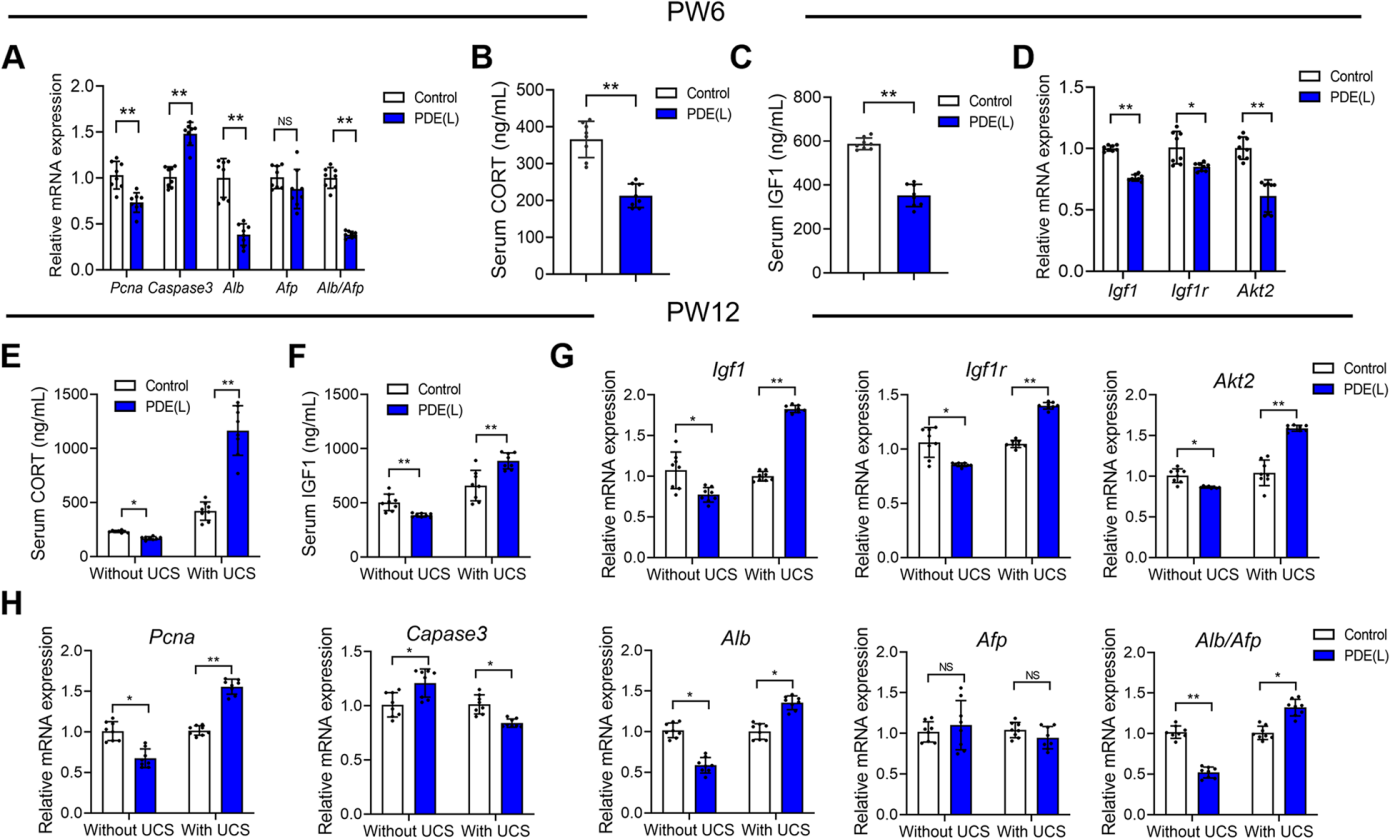
Effects of prenatal dexamethasone exposure (PDE) on postnatal liver development and the levels of corticosterone (CORT) and insulin-like growth factor 1(IGF1) at postnatal week (PW) 6 and 12. **A** The mRNA expression of liver development-related genes at PW6 (*n =* 8); (**B**) Serum CORT at PW6 (*n =* 8); (**C**) Serum IGF1 level at PW6 (*n =* 8); (**D**) The mRNA expression of liver IGF1 signaling pathway expression at PW6 (*n =* 8); (**E**) Serum CORT with or without unpredictable chronic stress (UCS) at PW12 (*n =* 8); (**F**) Serum IGF1 level with or without UCS at PW12 (*n =* 8); (**G**) The mRNA expression of liver IGF1 signaling pathway expression with or without UCS at PW12 (*n =* 8); (**H**) The mRNA expression of liver development-related genes with or without UCS at PW12 (*n =* 8). Mean ± S.E.M. ^*^ *P <* 0.05, ^**^ *P <* 0.01 vs. the control group. NS, no significance; PDE(L), prenatal dexamethasone exposure at a low dose (0.2 mg/kg∙d)

We then investigated how PDE affect liver development in the postnatal male offspring rats. The findings revealed that PDE reduced the expression of *Pcna* and increased the expression of *Caspase3* in the liver of male offspring rats at PW6 (*P <* 0.01; Fig. 4A). Additionally, it significantly decreased the expression of *Alb* and the expression ratio of *Alb* to *Afp* (*P <* 0.01; Fig. 4A). This suggests that PDE caused liver dysplasia and impaired liver function in postnatal male offspring rats. Moreover, PDE led to a continuous reduction in the serum corticosterone level (*P <* 0.01; Fig. 4B). Correspondingly, it significantly lowered the serum IGF1 level and suppressed the expression of IGF1 signaling pathway in the liver (*P <* 0.05, *P <* 0.01; Fig. 4C and D). Collectively, the diminished corticosterone and IGF1 expression were involved in the liver dysplasia observed in postnatal male offspring rats due to PDE.

Next, we analyzed the serum levels of corticosterone and IGF1, as well as the expression of liver IGF1 signaling pathway in the PDE offspring rats at PW12. The results showed that PDE reduced serum corticosterone levels in the offspring rats at PW12 (*P <* 0.05; Fig. 4E). Meanwhile, PDE led to a decrease in serum IGF1 level and reduced expression of the liver IGF1 signaling pathway (*P <* 0.05, *P <* 0.01; Fig. 4F and G). However, when these postnatal offspring rats experienced two weeks of unpredictable chronic stress (UCS) to elevate serum corticosterone level, both serum IGF1 level and liver IGF1 signaling pathway expression increased in alignment with the corticosterone elevation (*P <* 0.01; Fig. 4E-G). Moreover, PDE caused liver dysplasia and liver function impairment in these postnatal male offspring rats without UCS (*P <* 0.05, *P <* 0.01; Fig. 4H). However, when these postnatal offspring rats underwent two weeks of UCS to elevate serum corticosterone level, both liver dysplasia and liver function impairment were alleviated (*P <* 0.05, *P <* 0.01; Fig. 4H). These findings indicated that the low corticosterone level led to liver dysplasia in the postnatal male offspring rats due to PDE by downregulating IGF1 expression.

### The low corticosterone downregulated the H3K27 acetylation level of IGF1 and its expression ***via*** GRα/SP1/P300 signaling

**Fig. 5:**
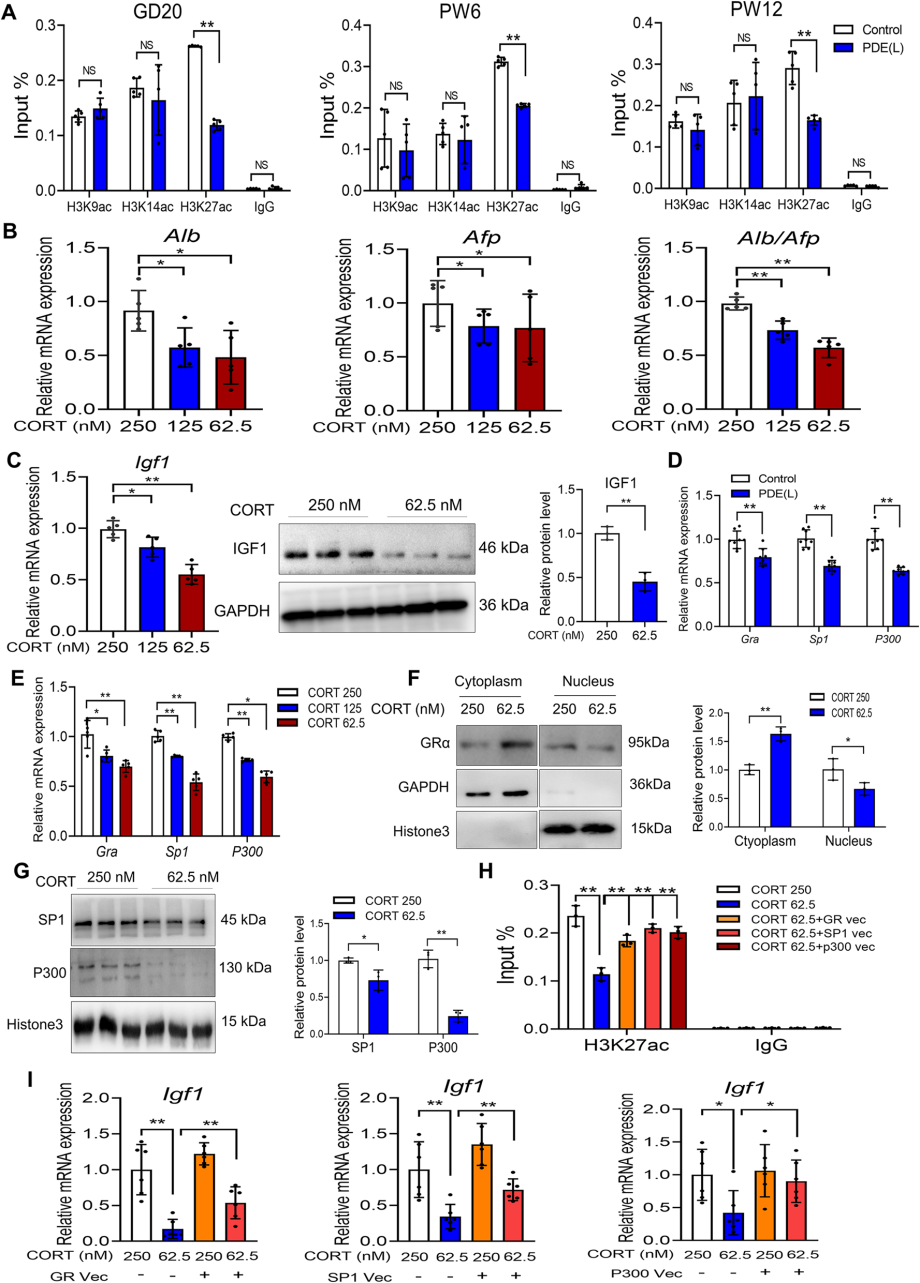
The specific epigenetic mechanism of low corticosterone (CORT) downregulating insulin-like growth factor I (IGF1) expression in vivo and in vitro. **A** The histone 3 lysine 9 acetylation (H3K9ac), H3K14ac, and H3K27ac levels of *Igf1* promoter region (*n =* 5); (**B**) The mRNA expression of albumin (*Alb*), and alpha-fetoprotein (*Afp*) (*n =* 5); (**C**) The mRNA expression (*n =* 5) and protein level (*n =* 3) of IGF1; (**D**) The mRNA expression of glucocorticoid receptor α (*Gra*), special protein 1 (*Sp1*), and *P300* in the fetal liver (*n =* 8); (**E**) The mRNA expression of *Grα*, *Sp1*, and *P300 in vitro* (*n =* 5); (**F**) The protein level of GRα in vitro (*n =* 3); (**G**) The protein level of SP1 and P300 in vitro (*n =* 3); (**H**) The H3K27ac level of *Igf1* promoter region (*n =* 3); (**I**) The *Igf1* mRNA expression with GR, SP1, and P300 overexpression plasmid (*n =* 6). Mean ± S.E.M. ^*^ *P <* 0.05, ^**^ *P <* 0.01 vs. the control group. NS, no significance

To uncover the specific epigenetic mechanism behind the downregulation of IGF1 expression due to low corticosterone, we investigated the acetylation levels of H3K9, H3K14, and H3K27 sites in the *Igf1* promoter region. We found that only the H3K27ac in the *Igf1* promoter region continued to decrease in both fetal and postnatal offspring rats due to PDE (*P <* 0.01; Fig. 5A). These results indicated that the reduced H3K27ac level in the *Igf1* promoter region played a role in its diminished expression. Furthermore, we established an hepatogenic cell differentiation model to confirm this molecular mechanism. The results showed that the low concentration of corticosterone inhibited the expression of hepatogenic differentiation marker genes in a concentration-dependent manner (*P <* 0.05, *P <* 0.01; Fig. 5B). Additionally, low corticosterone levels reduced the expression of *Igf1* during the hepatogenic differentiation of BMSCs (*P <* 0.05, *P <* 0.01; Fig. 5C). We also observed that the mRNA expression levels of *Gra*, *Sp1*, and *P300* were significantly downregulated by PDE in the fetal liver (*P <* 0.01; Fig. 5D). In vitro, the mRNA expressions of *Gra*, *Sp1*, and *P300* were similarly reduced by low corticosterone concentrations (*P <* 0.05, *P <* 0.01; Fig. 5E). Moreover, the low corticosterone inhibited the translocation of GRα into the nucleus and led to a reduction in SP1 and P300 protein levels in the nucleus of BMSCs (*P <* 0.05, *P <* 0.01; Fig. 5F and G). After overexpressing GRα, SP1, and P300, we found that these treatments significantly reversed the inhibitory effect of low corticosterone concentrations on the H3K27ac level of IGF1 and its expression (*P <* 0.05, *P <* 0.01; Fig. 5H and I). These results demonstrated that low corticosterone concentrations reduced the H3K27ac level of IGF1 and its expression by downregulating the GRα/SP1/P300 signaling pathway. This ultimately resulted in an inhibitory effect on hepatocyte differentiation.

### Clinical specimens verified the inhibited liver function of neonates caused by PDT due to the low serum cortisol and IGF1 levels

**Fig. 6:**
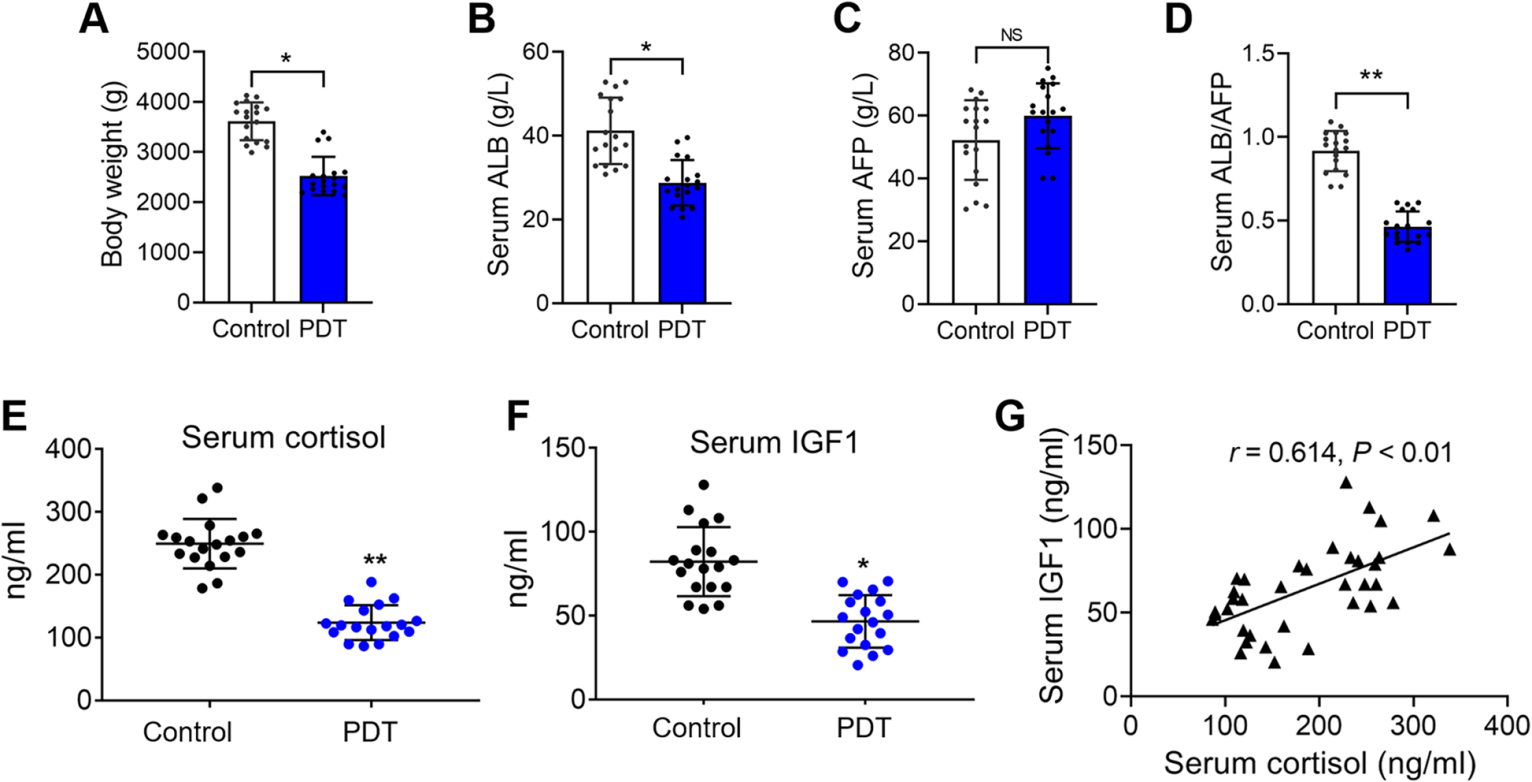
Effects of prenatal dexamethasone treatment (PDT) on the body weight, serum hepatic function markers, serum cortisol level, and serum insulin-like growth factor 1 (IGF1) level of the male neonates. **A** Neonatal body weight (*n =* 18); (**B**) Serum albumin (ALB) (*n =* 18); (**C**) Serum alpha-fetoprotein (AFP) (*n =* 18); (**D**) The ratio of ALB to AFP (*n =* 18); (**E**) Serum cortisol level (*n =* 18); (**F**) Serum IGF1 level (*n =* 18); (**G**) The correlation analysis between serum cortisol and IGF1; Mean ± S.E.M. ^*^ *P <* 0.05, ^**^ *P <* 0.01 vs. the control group. NS, no significance

Finally, we conducted a statistical analysis of male neonates’ weight from both the control and PDT groups. The results revealed that PDT led to a reduction in the birth weight of male neonates (*P <* 0.05; Fig. 6A). Additionally, we observed a significant decrease in umbilical cord serum ALB levels due to PDT, while serum AFP levels were not notably affected by PDT (*P <* 0.05; Fig. 6B). Although there was no significant alteration in serum AFP levels (Fig. 6C), the ratio of ALB/AFP was significantly decreased by PDT (*P <* 0.01; Fig. 6D). These findings align with the observed impact on liver function in low birth weight neonates, consistent with our animal experiments. Furthermore, the umbilical cord serum cortisol and IGF1 levels in male neonates who received dexamethasone treatment were significantly lower than those in the control group (*P <* 0.05, *P <* 0.01; Fig. 6E and F). Correlation analysis indicated a positive relationship between serum cortisol levels and serum IGF1 levels (*P <* 0.01; Fig. 6G). These results confirm that the diminished liver function in neonates resulting from PDT is associated with reduced serum cortisol and IGF1 levels.

## Discussion

### The insulin resistance and metabolic syndrome observed in male offspring due to PDE traced back to fetal liver dysplasia

The prevalence of metabolic syndrome is on the rise, particularly among younger populations, with estimates ranging from 20 to 40% in various populations [29]. While postnatal lifestyle factors contribute significantly to the development of metabolic syndrome, it is increasingly evident that an individual’s risk for this condition is also influenced by adverse conditions during pregnancy [30]. Researches have linked exposure to adverse conditions in utero with an increased risk of metabolic syndrome, type 2 diabetes, and cardiovascular diseases in adulthood [31, 32]. Dexamethasone treatment during pregnancy is a known adverse factor during pregnancy for fetuses and also affects maternal metabolism at the peripartum [9, 10, 33, 34]. In this study, pregnant rats were treated with dexamethasone (0.2 and 0.8 mg/kg·d) during middle to late pregnancy to investigate the effects of PDE on metabolic syndrome and its developmental origins. Dexamethasone is commonly administered to pregnant women at risk of preterm birth between 24 and 37 weeks of gestation to reduce the incidence of respiratory distress syndrome [35, 36]. Additionally, prolonged dexamethasone exposure during pregnancy is common due to challenges in early preterm birth diagnosis [37, 38]. Therefore, the dose and timing of dexamethasone treatment in this study were chosen to investigate the phenomenon and mechanism of the developmental origins of metabolic syndrome.

The study found that PDE led to increased serum glucose levels and induced hyperlipidemia and hypercholesterolemia in adult male offspring, characterized by elevated serum TG and TCH levels and reduced HDL-C levels. The study also observed lipid deposition in various organs of adult male offspring, including the liver, bone, adrenal gland, kidney, and pancreas. Furthermore, PDE resulted in insulin resistance in these male adult offspring rats. Therefore, the study systematically demonstrated that PDE induced insulin resistance and metabolic syndrome in adult male offspring rats. These phenomena were also reported previously by Pantaleão et al. [39]. We also observed a mild susceptibility to metabolic syndrome in female adult offspring due to PDE (Fig. S1), though they did not exhibit the typical symptoms seen in male offspring. Previous studies have shown that male offspring experienced hypertension in response to dexamethasone exposure [40]. It has also been demonstrated that maternal ethanol exposure led to more severe glucose intolerance and insulin insensitivity in male offspring rats [41]. These gender differences align with the current study’s findings. Therefore, for further exploration of the developmental origins of metabolic syndrome, male offspring rats were selected in this study.

The liver is an insulin-sensitive organ that plays a crucial role in regulating overall energy balance in the body [42]. It is the largest metabolic organ and is involved in glucose and lipid metabolism. Exposure to exogenous chemical factors during pregnancy can have toxic effects on the development of offspring before birth, resulting in growth retardation, functional impairments, and structural abnormalities [15, 43]. This study demonstrated that PDE led to liver dysplasia and impaired liver function in both fetal and postnatal male offspring rats. This suggests that the susceptibility to metabolic syndrome induced by PDE originated from intrauterine liver dysplasia.

### The low endogenous glucocorticoid downregulating IGF1 expression contributed to PDE-induced liver dysplasia in offspring

Intrauterine basal levels of endogenous glucocorticoids play a critical role in regulating fetal tissue differentiation and functional maturation. However, low concentrations of endogenous glucocorticoids in the fetus can lead to developmental abnormalities, such as IUGR [23]. Moreover, low levels of endogenous glucocorticoids during the fetal period, caused by adverse prenatal conditions, can contribute to dysplasia in multiple organs [44]. Throughout fetal development, IGF1 is primarily produced by the fetal liver. It plays a vital role in determining fetal birth weight and organ development by promoting the enrichment and functional differentiation of stem cells [21]. Previous research has shown that IUGR fetuses exhibit reduced serum IGF1 levels, and knocking down liver IGF1 significantly reduces fetal birth weight and body length [45].

This study revealed that exposure to dexamethasone during pregnancy resulted in reduced blood corticosterone levels in both fetal and postnatal male offspring rats. These results were also reported previously by Almeida et al. [46]. Additionally, the serum IGF1 level and the expression of the IGF1 signaling pathway in the liver were significantly downregulated in male offspring exposed to dexamethasone. However, when these postnatal male offspring rats experienced two weeks of chronic stress, which elevated serum corticosterone levels, both serum IGF1 levels and liver IGF1 signaling pathway expression increased. Furthermore, liver dysplasia and liver function inhibition were partially rescued by this chronic stress. In vitro experiments also demonstrated that low concentrations of corticosterone reduced the expression of IGF1 during the hepatogenic differentiation of BMSCs. Remarkably, the study confirmed that the reduced liver function in neonates caused by PDT was closely associated with low serum cortisol and IGF1 levels. This finding aligns with the results obtained from animal and cell experiments. In summary, these results suggest that low endogenous glucocorticoid levels contribute to the PDE-induced liver dysplasia in offspring by downregulating IGF1 expression. However, the results of this study are based on animal models, in vitro cell differentiation models, and validation using samples from a small clinical population. Currently, there is still a lack of sufficient validation from large human population data, which is a limitation of this research. It is also one of the research aspects that need to be further addressed and expanded in the later stages.

### The low endogenous glucocorticoid downregulated the H3K27 acetylation level of IGF1 and its expression *via* GRα/SP1/p300 signal

Epigenetic modifications involve changes in heritable gene expression without alterations in the DNA sequence. This can encompass histone acetylation modifications, a normal process in mammalian development. However, abnormalities in histone acetylation have been linked to the development of diseases that originate during early development [13, 47]. Glucocorticoids primarily activate the GR and recruit histone acetylase and transcription factors. Together, they regulate the histone acetylation levels in the promoter regions of target genes [48].

Therefore, when investigating the epigenetic regulatory mechanisms responsible for the reduced IGF1 expression resulting from low corticosterone concentrations, we examined the histone acetylation levels in the *Igf1* promoter region. Our findings indicate that only the acetylation levels of H3K27 sites in the *Igf1* promoter region consistently decreased in fetal and postnatal offspring rats exposed to PDE. Furthermore, we observed downregulation of expressions of *Gra, Sp1*, and *P300* in response to low corticosterone concentrations. In an in vitro setting, low concentrations of corticosterone inhibited the translocation of GRα into the nucleus and led to reduced SP1 and P300 protein levels in the nucleus of BMSCs. However, after overexpressing GRα, SP1, and P300, these treatments effectively reversed the inhibitory effects of low corticosterone concentrations on H3K27ac levels in the *Igf1* promoter region and its expression. These results suggest that low corticosterone concentrations reduce the H3K27ac levels in the *Igf1* promoter region and its expression by downregulating the GRα/SP1/P300 signaling pathway, resulting in impaired hepatocyte differentiation.

## Conclusions

**Fig. 7:**
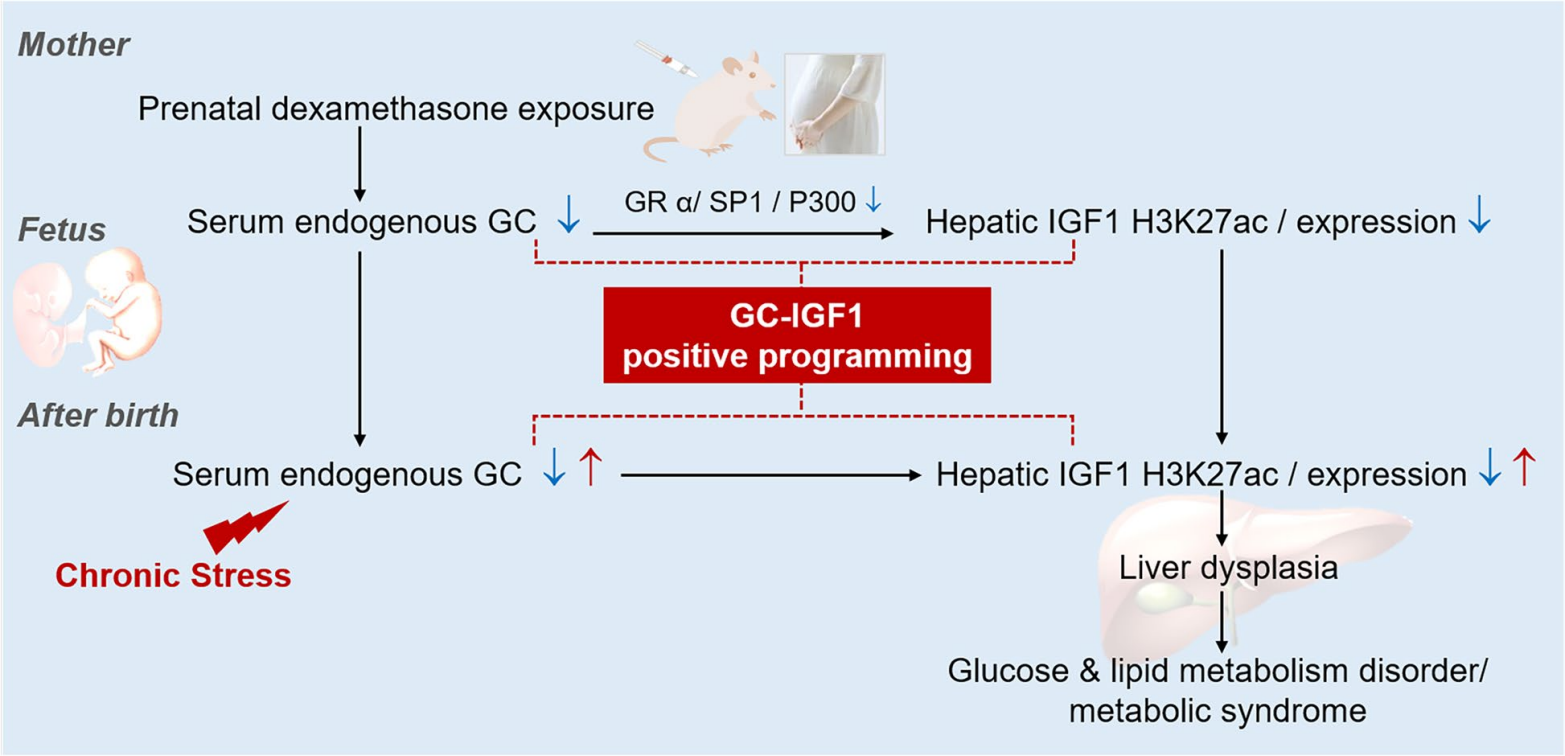
Dexamethasone exposure during pregnancy triggers metabolic syndrome in offspring *via* epigenetic alteration of IGF1. GC, glucocorticoid; GRα, glucocorticoid receptor α; SP1, special protein 1; IGF1, insulin-like growth factor 1; H3K27ac, histone 3 lysine 27 acetylation

In summary (Fig. 7), our study demonstrated that PDE is responsible for inducing insulin resistance and metabolic syndrome in adult male offspring, with its origins traced back to intrauterine liver dysplasia. We have further demonstrated that low concentrations of corticosterone lead to reduced H3K27ac levels in the IGF1 promoter region and its expression by downregulating the GRα/SP1/P300 signaling pathway, ultimately resulting in the inhibition of hepatocyte differentiation and liver dysplasia. In a clinical context, our analysis of serum specimens verified that the compromised liver function in neonates exposed to dexamethasone is closely linked to low serum cortisol and IGF1 levels. This research provides an innovative understanding of the developmental origin of metabolic syndrome in adults due to PDE. These insights have important theoretical and practical implications, guiding the rational use of dexamethasone during pregnancy and contributing to the development of early prevention and treatment strategies for metabolic syndrome.

### Supplementary Information

**Additional file 1: Table S1. **Oligonucleotide primers and PCR conditions of rat in real-time quantitative PCR. **Fig. S1.** Effects of prenatal dexamethasone exposure (PDE) on glucose and lipid metabolism in the adult female offspring rats at postnatal week 28. A. Serum glucose level; B. Serum triglyceride (TG) level; C. Serum total cholesterol (TCH) level; D. Serum high-density lipoprotein- cholesterol (HDL-C) level; E. Serum low-density lipoprotein-cholesterol (LDL-C) level; F. The ratio of TG to HDL-C; G. The ratio of TCH to HDL-C; H. The ratio of LDL-C to HDL-C; I. Serum insulin level; J. Insulin resistance index (IRI). ^*^P < 0.05; ^**^P < 0.01; the control group *vs.* the PDE group. *n *= 8 per group. PDE (L), prenatal dexamethasone exposure at a low dose (0.2 mg/kg∙d).

## Data Availability

The datasets used and analyzed during the current study are available from the corresponding author on reasonable request.

## Abbreviations

PDE: Prenatal dexamethasone exposure
ALB: Albumin
AFP: Alpha-fetoprotein
IGF1: Insulin-like growth factor 1
ELISA: Enzyme-linked immunosorbent assay
GRα: Glucocorticoid receptor α
SP1: Special protein 1
GAPDH: Glyceraldehyde-3-phosphate dehydrogenase
H3K27ac: Histone 3 lysine 27 acetylation
GD: Gestational day
IUGR: Intrauterine growth retardation
PD: Postnatal day
PW: Postnatal week
IPGTT: Intraperitoneal glucose tolerance test
ITT: Insulin tolerance test
BMSCs: Bone marrow mesenchymal stem cells
IRI: Insulin resistance index
UCS: Unpredictable chronic stress
PDT: Prenatal dexamethasone treatment
TCH: Total cholesterol
TG: Triglyceride
LDL-C: Low-density lipoprotein-cholesterol
HDL-C: High-density lipoprotein-cholesterol
IHC: Immunohistochemical
ChIP: Chromatin immunoprecipitation
PCNA: Proliferating cell nuclear antigen
