# Supplementary material for: Dexamethasone exposure during pregnancy triggers metabolic syndrome in offspring via epigenetic alteration of IGF1
Source: Cell Commun Signal. 2024 Jan 23;22:62. doi: 10.1186/s12964-024-01472-6 (PMC10807214; doi:10.1186/s12964-024-01472-6)
Supplement: Supplementary file 1 — Additional file 1: Table S1. Oligonucleotide primers and PCR conditions of rat in real-time quantitative PCR. Fig. S1. Effects of prenatal dexamethasone exposure (PDE) on glucose and lipid metabolism in the adult female offspring rats at postnatal week 28. A. Serum glucose level; B. Serum triglyceride (TG) level; C. Serum total cholesterol (TCH) level; D. Serum high-density lipoprotein- cholesterol (HDL-C) level; E. Serum low-density lipoprotein-cholesterol (LDL-C) level; F. The ratio of TG to HDL-C; G. The ratio of TCH to HDL-C; H. The ratio of LDL-C to HDL-C; I. Serum insulin level; J. Insulin resistance index (IRI). *P < 0.05; **P < 0.01; the control group vs. the PDE group. n = 8 per group. PDE (L), prenatal dexamethasone exposure at a low dose (0.2 mg/kg∙d). [file 12964_2024_1472_MOESM1_ESM.pdf]

**Dexamethasone Exposure during Pregnancy Triggers Metabolic Syndrome in Offspring *via* Epigenetic Alteration of IGF1**

Hao Xiao ^1,2,3,#^, Bo He ^1,#^, Heze Liu ^1,#^, Yawen Chen ^1^, Di Xiao ^1^, Hui Wang ^1,3*^

^1^ Department of Pharmacology, Wuhan University School of Basic Medical Sciences, Wuhan, 430071, China;

^2^ Division of Joint Surgery and Sports Medicine, Department of Orthopedic Surgery, Zhongnan Hospital of Wuhan University, Wuhan, 430071, China;

^3^ Hubei Provincial Key Laboratory of Developmentally Originated Disease, Wuhan,430071, China.

**^*^Corresponding author:**

Hui Wang, Ph.D., Department of Pharmacology, Wuhan University School of Basic Medical Sciences. Tel: +86-13627232557; E-mail: [wanghui19@whu.edu.cn](mailto:wanghui19@whu.edu.cn).

^#^These authors contributed equally to this research.

**Table S1. Oligonucleotide primers and PCR conditions of rat in real-time quantitative PCR.**

| Genes | Forward primer | Reverse primer |
| --- | --- | --- |
| *Gra* | CACCCATGACCCTGTCAGTC | AAAGCCTCCCTCTGCTAACC |
| *Sp1* | GGGATCAGCTCCTTGCACTCT | CCAGGGATCACTGTCAAAAACA |
| *P300* | AGATTCAGAGGGCAGCAGAGAC | GCCATAGGAGGTGGGTTCATAC |
| *Igf1* | TCAGTTCGTGTGTGGACCAG | TCACAGCTCCGGAAGCAAC |
| *Igf1r* | GTCCTTCGGGATGGTCTA | TGGCCTTGGGATACTACAC |
| *Akt-2* | TGCTACGCTTACGCACCTGCC | AGAGAGCTCAGGGCAGCAGGAC |
| *Pcna* | GGGCTGAAGATAATGCTGATACC | ATGTTCCCATTGCCAAGCTC |
| *Caspase-3* | GAACGAACGGACCTGTGGAC | AGTAACCGGGTGCGGTAGAG |
| *Alb* | CGCCCATCGGTTTAAGGACT | ACACTCGTTTCTTTCGGGCT |
| *Afp* | CACTGGCGATGGGTGTTTAG | GTCTGGAGCGGTCTTCTTGC |
| *Gapdh* | GGCACAGTCAAGGCTGAGAATG | ATGGTGGTGAAGACGCCAGTA |

*Gra*, glucocorticoid receptor α; *Sp1*, special protein 1; *Igf1*, insulin-like growth factor 1; *Igf1r*, insulin-like growth factor-1 receptor; *Pcna*, proliferating cell nuclear antigen; *Caspase-3*, cysteine-containing aspartate-specific protease-3; *Alb*, albumin; *Afp*, alpha-fetoprotein; *Gapdh*, glyceraldehyde phosphate dehydrogenase.


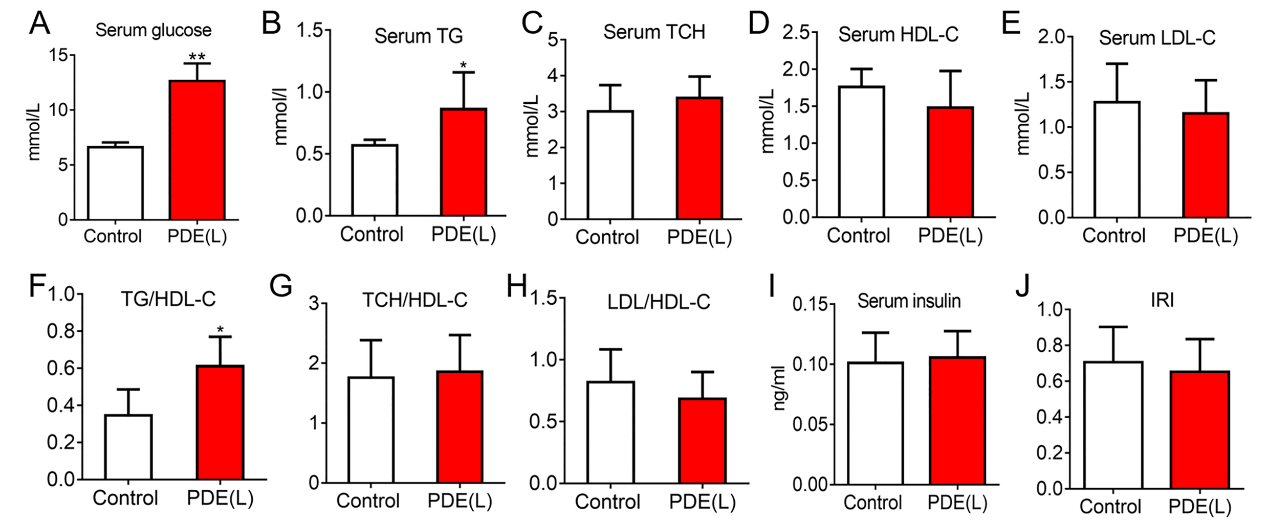


**Fig.S1. Effects of prenatal dexamethasone exposure (PDE) on glucose and lipid metabolism in the adult female offspring rats at postnatal week 28.** A. Serum glucose level; B. Serum triglyceride (TG) level; C. Serum total cholesterol (TCH) level; D. Serum high-density lipoprotein- cholesterol (HDL-C) level; E. Serum low-density lipoprotein-cholesterol (LDL-C) level; F. The ratio of TG to HDL-C; G. The ratio of TCH to HDL-C; H. The ratio of LDL-C to HDL-C; I. Serum insulin level; J. Insulin resistance index (IRI). ^*^*P* < 0.05; ^**^*P* < 0.01; the control group *vs.* the PDE group. *n* = 8 per group. PDE (L), prenatal dexamethasone exposure at a low dose (0.2 mg/kg∙d).
